# Resveratrol-Induced Downregulation of NAF-1 Enhances the Sensitivity of Pancreatic Cancer Cells to Gemcitabine via the ROS/Nrf2 Signaling Pathways

**DOI:** 10.1155/2018/9482018

**Published:** 2018-03-22

**Authors:** Liang Cheng, Bin Yan, Ke Chen, Zhengdong Jiang, Cancan Zhou, Junyu Cao, Weikun Qian, Jie Li, Liankang Sun, Jiguang Ma, Qingyong Ma, Huanchen Sha

**Affiliations:** ^1^Department of Hepatobiliary Surgery, First Affiliated Hospital of Xi'an Jiaotong University, Xi'an 710061, China; ^2^Department of Anesthesiology, First Affiliated Hospital of Xi'an Jiaotong University, Xi'an 710061, China

## Abstract

NAF-1 (nutrient-deprivation autophagy factor-1), which is an outer mitochondrial membrane protein, is known to play important roles in calcium metabolism, antiapoptosis, and antiautophagy. Resveratrol, a natural polyphenolic compound, is considered as a potent anticancer agent. Nevertheless, the molecular mechanisms underlying the effects of resveratrol and NAF-1 and their mediation of drug resistance in pancreatic cancer remain unclear. Here, we demonstrate that resveratrol suppresses the expression of NAF-1 in pancreatic cancer cells by inducing cellular reactive oxygen species (ROS) accumulation and activating Nrf2 signaling. In addition, the knockdown of NAF-1 activates apoptosis and impedes the proliferation of pancreatic cancer cells. More importantly, the targeting of NAF-1 by resveratrol can improve the sensitivity of pancreatic cancer cells to gemcitabine. These results highlight the significance of strategies that target NAF-1, which may enhance the efficacy of gemcitabine in pancreatic cancer therapy.

## 1. Introduction

Pancreatic ductal adenocarcinoma (PDAC) is the most lethal and malignant type of cancer and causes more than 43,000 estimated deaths per year in the United States. The overall five-year survival rate is less than 7% [1]. Pancreatic cancer is predicted to be the second leading cause of cancer-associated mortality within the next 5–10 years [2], which is partially due to the lack of early diagnosis and effective treatment options. Currently, the therapeutic options for pancreatic cancer remain very limited. Therefore, it is important to identify additional new therapeutic tools for early disease detection and the development of therapeutic biomarkers and strategies [3].

The protein nutrient-deprivation autophagy factor-1(NAF-1), which is encoded by the CISD2 gene, is located in the outer mitochondrial membrane, endoplasmic reticulum (ER), and mitochondria-associated membrane (MAM) members [4]. NAF-1 contains a transmembrane domain that consists of a conserved amino acid sequence for iron binding, namely, the CDGSH domain [5], which mainly mediates the mitochondrial integrity and lifespan in mammals [6]. It has been reported that the mRNA expression level of NAF-1 is decreased in older mice compared with that in younger mice [7]. Currently, it is recognized that cancer cells acquire a long lifespan, which is a benefit to their unlimited growth capacity. Therefore, it is hypothesized that NAF-1 plays an important role in tumor cells, actively participates in clinical therapy, and exerts a beneficial effect on the prognosis of patients. In addition, NAF-1 serves as an important regulator by antagonizing the BECN1-mediated cellular autophagy at the endoplasmic reticulum [8] and is required for the BCL2-mediated depression of endoplasmic reticulum Ca^2+^ storage during autophagy [9, 10]. Therefore, a deficiency in NAF-1 leads to mitochondrial damage and induces autophagy to eliminate the dysfunctional organelles [4]. Recently, it has been proposed that the expression of NAF-1 may act as a novel biomarker that is correlated with pelvic lymph node metastasis and prognosis in patients with early-stage cancer [10] and the overexpression of NAF-1 prevents human epithelial breast cancer cells from significantly reducing cell proliferation and tumor growth. In contrast, apoptosis is activated in NAF-1-deficient human epithelial breast cancer cells [11]. However, the role of NAF-1 in pancreatic cancer therapy remains unknown. The abovementioned observations suggested that modulating NAF-1 could have a positive effect on pancreatic cancer therapy and improve prognosis.

Resveratrol (RSV, trans-3,5,4-trihydroxystilbene), which is a natural polyphenolic compound, is found in grapes, peanuts, a variety of berries, and food products, such as red wine [12]. Resveratrol is widely recognized as a natural antioxidant, which possesses the ability to lower the level of ROS both in cancer [13] and in noncancerous disease [14]. Through the ROS-scavenging property, resveratrol may reduce oxidative stress-induced apoptosis, cellular aging, and cell death [15], thus presenting a protective effect for cells against unfavorable environment. On the contrary, upregulated ROS production is also found in resveratrol-treated cells, which causes apoptosis and autophagy [16] and therefore induces cytotoxic effects. Resveratrol has been analyzed as a protective or therapeutic agent in pancreatic cancer models and was shown to exert a synergistic antitumor effect with gemcitabine [17]. Resveratrol has a variety of pharmacological functions, such as anti-inflammation and antioxidant activities [18–20], inhibiting tumor growth, invasion, and the epithelial-mesenchymal transition [21]. In recent years, people pay more attention to the antioxidant activities of resveratrol and ignore the prooxidant activity in cancer cell. Many other in vitro and clinical studies have been conducted involving anticancer activity of resveratrol via increasing intracellular reactive oxygen species (ROS) production [22]. For example, W. Lee and D. G Lee have recently reported that resveratrol could induce membrane and DNA disruption via its prooxidant activity in cancer cells [23]. However, it is unclear whether resveratrol could impact the biological characteristics of tumors via other molecules. According to the previous studies, resveratrol and the antidiabetic drug pioglitazone can abrogate the ability of NAF-1 to transfer the cluster of acceptor proteins and iron to mitochondria [24]. These results reveal that resveratrol could prevent pancreatic cancer cells from growing via NAF-1 signaling. However, the molecular mechanism underlying the effects of resveratrol and NAF-1 has not been elucidated. In this article, we aim to explore the effect of resveratrol on the expression of NAF-1 and clarify its mechanism.

Multiple signaling pathways have been implicated in protecting cell from ROS overproduction caused oxidative stress. Nrf2 is a redox-sensitive transcriptional factor and usually upregulated in various cancers. But the specific role of Nrf2 in cancer still remains controversial, since Nrf2 is considered both as a tumor suppressor and a tumor promoter. Some researchers postulate that its precise role is dependent on the stage of tumorigenesis [25, 26], which leads to the question of whether Nrf2 should be targeted for anticancer therapeutic approaches. Nrf2 often exists in the cytoplasm as an inactive state via tethering to Kelch-like ECH-associated protein (Keap1) and could be activated by oxidative stress [27]. After which, Nrf2 dissociates with the repressor protein Keap1 and translocates into the nucleus, interacting with antioxidant response elements (ARE). As a result, the subsequent expression of numerous downstream genes relating with redox homeostasis are induced [28, 29]. Chen et al. [30] have reported that Nrf2 and several antioxidant enzyme expression were upregulated in cells following BDE-47 treatment which induces the intracellular ROS accumulation. In our previous study, we have showed that the Nrf2 pathway and autophagy were activated by ROS in pancreatic cancer cells [31]. Moreover, the Nrf2 pathway can antagonize cellular stress signals by promoting a series of antioxidant programs upon ROS stimulation. However, more recent data reveal its role also as a tumor suppressor [32, 33]. For example, Probst et al. [34] found that genetic activation of Nrf2 and pharmacological activation of Nrf2 by RTA 405 are distinct, which suppress cancer cell survival and promote apoptosis. Resveratrol, the same as an antioxidant inflammation modulator, may have the same pharmacology action and mechanism and promotes the apoptosis of pancreatic cancer cells via activation of Nrf2 pathway. In this study, we aimed to explore the role of Nrf2 as a tumor suppressor in pancreatic cancer cells.

NAF-1 serves as an important regulator by antagonizing the BECN1-mediated cellular autophagy at the endoplasmic reticulum [17] and takes a close relationship with apoptosis. Therefore, we speculated that the activation of Nrf2 pathway induced by resveratrol promotes apoptosis of pancreatic cancer cells through inhibition of the downregulation of NAF-1. And finally, we aimed to clarify their role in improving the sensitivity of pancreatic cancer cells to gemcitabine.

In this study, we demonstrated that resveratrol could activate Nrf2 and suppress the expression of NAF-1 in pancreatic cancer cells by inducing the accumulation of ROS. More importantly, decreasing the expression of NAF-1 impeded the proliferation of and activated apoptosis in pancreatic cancer cells. The targeting of NAF-1 via resveratrol can enhance the sensitivity of pancreatic cancer cells to gemcitabine. Optimistically, we further explored the role of NAF-1 as a novel molecular target for improving the efficacy of the currently used chemotherapeutic regimens in patients with pancreatic cancer and improving their clinical prognosis.

## 2. Materials and Methods

All experimental protocols were approved by the Ethical Committee of the First Affiliated Hospital of Medical College, Xi'an Jiaotong University, Xi'an, China.

### 2.1. Reagents and Antibodies

Resveratrol (>99% pure), MTT (3-(4,5-dimethyl-2-thiazolyl)-2,5-diphenyl-2-H-tetrazolium bromide), and NAC (N-acetyl-L-cysteine) were purchased from Sigma-Aldrich (St. Louis, MO, USA), and gemcitabine was purchased from Selleck Chemicals (Houston, TX, USA). Analytical grade 30% H_2_O_2_ was obtained from Sinopharm Chemical Reagent Co. Ltd. (Shanghai, China). DCFH-DA was obtained from the Beyotime Institute of Biotechnology (Haimen, China). The antibodies used in this study against NAF-1 and *α*-tubulin were purchased from Proteintech Group (Chicago, IL). The primary antibodies against Nrf2, Bax, and Bcl-2 were obtained from Abcam (Cambridge, MA, USA). Resveratrol and gemcitabine were initially dissolved in dimethyl sulfoxide (DMSO) at stock concentrations of 50 mM and 10 mM, respectively. Working dilutions of resveratrol and gemcitabine were prepared in a culture medium immediately before use, and DMSO was used as the control in all experiments.

### 2.2. Cell Lines and Cell Culture

The human pancreatic cancer cell lines Panc-1, Mia paca-2, CF pac-1, and BxPC-3 were purchased from the Type Culture Collection of the Chinese Academy of Sciences (Shanghai, China) and cultured as described previously [17]. In brief, Panc-1 and Mia paca-2 were cultured in a humidified atmosphere containing 5% CO_2_ and 95% air at 37°C in Dulbecco's Modified Eagle Medium (DMEM), containing 10% FBS (HyClone, Logan, UT, USA), 100 U/mL penicillin, and 100 *μ*g/mL streptomycin.

### 2.3. Western Blot Analysis

**Figure 1:**
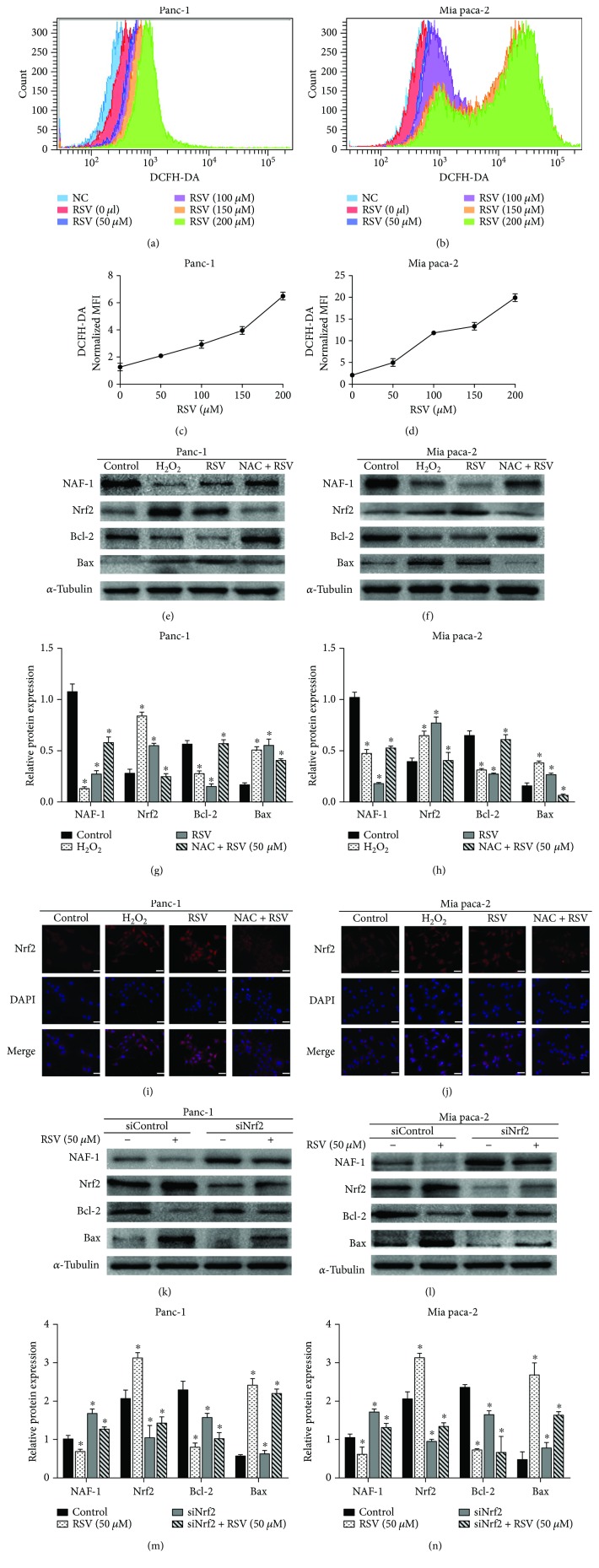
Resveratrol suppresses the level of NAF-1 and enhances the expression of Nrf2 by inducing the accumulation of ROS, which contributed to cell death in the Panc-1 and Mia paca-2 cells. (a–d) The pancreatic cancer cells were treated with increasing doses of RSV (0, 50, 100, 150, and 200 *μ*M) for 24 h, and the accumulation of ROS was detected using 2′, 7′-dichlorofluorescin diacetate (DCFH-DA) probes. Representative flow cytometric pictures showing the mean fluorescence intensity (MFI) in each group were generated to create a composite image. (e–h) Indicated groups of pancreatic cancer cells were treated with RSV, H_2_O_2_, and NAC. Western blotting was performed to detect NAF-1, Nrf2, and the apoptosis-related proteins (Bax and Bcl-2). (i, j) Immunofluorescence staining of Nrf2 showed translocation of Nrf2 under the resveratrol and ROS stimulation in Panc-1 and Mia paca-2 cells (magnification, 400x; scale bar, 20 *μ*m). (k–n) After the transfection with siNrf2 or siControl for 48 h, the Panc-1 and Mia paca-2 cells were treated with resveratrol for 24 h, and the protein levels of NAF-1, Nrf2, and the apoptosis-related proteins (Bax and Bcl-2) were detected by Western blotting. All the images represent the results of three independent experiments. Quantified histograms of the Western blots were shown alongside. Column: mean; bar: SD. ^∗^ *P* < 0.05 compared with NAF-1 control group.

To evaluate the protein expression of NAF-1, Nrf2, Bax, Bcl-2, and *α*-tubulin in pancreatic cell lines, the semiquantitative densitometric analysis was performed. The experimental procedures were described previously [17]. In short, the total proteins were lysed using RIPA lysis buffer (Beyotime, Guangzhou, China), and the concentration of the proteins was determined by a BCA protein assay kit (Pierce, Rockford, IL, USA). Briefly, the proteins were separated on SDS-PAGE gel after the proteins were transferred to polyvinylidene difluoride (PVDF) membranes. The membranes were blocked with 5% fat-free milk for 2 h and then incubated with the primary antibodies (listed in Supplementary Material Table S1) at 4°C overnight. At last, the membranes were immunoblotted with a secondary antibody (diluted 1 : 10,000) for 2 h at 37°C. The probed proteins were detected on an enhanced chemiluminescence (ECL) PLUS system and a Molecular Imager ChemiDoc XRS System (Bio-Rad Laboratories, Hercules, CA, USA). All the bands in the same image such as Figures 1(e) and 1(f) are derived from the same blot, respectively, and all the data represent the results of three independent experiments.

### 2.4. Real-Time PCR

Total RNA was extracted using the Fastgen1000 RNA isolation system (Fastgen, Shanghai, China) according to the manufacturer's protocol. Total RNA was reverse-transcribed into cDNA using the Prime Script RT Reagent Kit (TaKaRa, Dalian, China). Real-time PCR was used to quantitatively examine the expression of NAF-1 and Nrf2 at the mRNA level. The PCR primer sequences for NAF-1 and Nrf2 are shown in Supplementary Material Table S3. The real-time PCR was conducted using the CFX Manager 2.1 fluorescent quantitative PCR kit (Bio-Rad Laboratories, Hercules, CA, USA) under the following conditions: 10 min at 95°C, followed by 40 cycles at 95°C for 2 sec, 60°C for 20 sec, and 70°C for 10 sec. Following the qPCR, a dissociation curve analysis was conducted. The expression of the target genes was determined using *α*-tubulin as the internal control. The relative gene expression level was calculated using the 2^−∆∆Ct^ method [35].

### 2.5. Immunofluorescence Staining

The cells for fluorescent immunocytochemistry were fixed for 15 min in 4% formaldehyde diluted in phosphate-buffered saline (PBS), permeabilized with 0.3% Triton X-100 for 10 min, and treated with blocking buffer (5% BSA in PBS) for 1 h at room temperature. Cells were incubated with primary antibody overnight at 4°C. Finally, the cells were washed and incubated with the red or green lgG antibody from Jackson Immunoresearch Laboratories (West Grove, PA, USA) for 1 h at room temperature. The cells were examined under a Zeiss instrument confocal microscope.

### 2.6. Viability Assay

Cell viability assay was conducted according to a previous report [36]. Briefly, Panc-1 and Mia paca-2 cells were seeded in 96-well plates at a density of 5000 cells/well and treated with various concentrations (0, 25, 50, 100, and 200 *μ*M) of resveratrol and different concentrations (0, 1, 2, 5, 10, and 20 *μ*M) of gemcitabine for 24, 48, and 72 h. The cell viability was assessed by the MTT assay. 15 *μ*L of 5 mg/mL MTT was added to the well, and then the mixture was incubated at 37°C for 4 h. Then, 100 *μ*L of DMSO was added to each well, and the optical density (OD) at 490 nm was measured using a multifunction microplate reader (POLAR star OPTIMA; BMG, Offenburg, Germany).

### 2.7. Apoptosis Assay

Apoptosis was evaluated by an Annexin V-FITC/PI apoptosis detection kit according as described previously [37] Briefly, the cancer cells were seeded in 6-well plates at a density of 1 × 10^5^ cells per well, and each treatment was applied for 48 h after the medium was removed. Then, cells were washed with PBS and stained with the FACSCalibur flow cytometry (BD Biosciences, San Diego, CA, USA).

### 2.8. Colony Formation Assay

One thousand cells were plated in a 35 mm Petri dish after a different treatment was applied for 24 h and allowed to grow for two weeks. The medium was changed twice per week. Then, the colonies were fixed with 4% paraformaldehyde, stained with a 0.1% crystal violet solution for 10 min, rinsed, and then imaged. The number of colonies (>0.5 mm) was calculated by a microscope (Nikon Eclipse Ti-S, Tokyo, Japan) at a magnification.

### 2.9. Measurement of Intracellular ROS

The intracellular ROS was detected by the ROS assay kit. In brief, after the media were removed and the wells were washes with PBS twice, 10 *μ*M of 2′-7′-dichlorodi-hydrofluorescein diacetate (DCFH-DA) was added to each well. Then, the cells were incubated in the dark at 37°C for 30 min. After washing twice with PBS and trypsinization, the cells were collected and immediately analyzed by flow cytometry using a FACSCalibur (BD Biosciences, San Diego, CA, USA) instrument.

### 2.10. Gene Silencing by Small Interfering RNA

siRNAs targeting NAF-1 and Nrf2 were purchased from GenePharm (Shanghai, China). The siRNA sequences are provided in Supplementary Material Table S2. Each siRNA (100 nM) was transfected into cells using Lipofectamine 2000 (Invitrogen, CA, USA) according to the manufacturer's instructions. The effect of each target gene was confirmed.

### 2.11. Statistical Analysis

Each experiment was independently performed at least three times. Data are presented as means ± standard deviation. Differences were evaluated using Student's *t*-test, with *p* < 0.05 considered to be statistically significant.

## 3. Results

### 3.1. The Expression Levels of NAF-1 and Nrf2 in Four Different Pancreatic Cancer Cells

**Figure 2:**
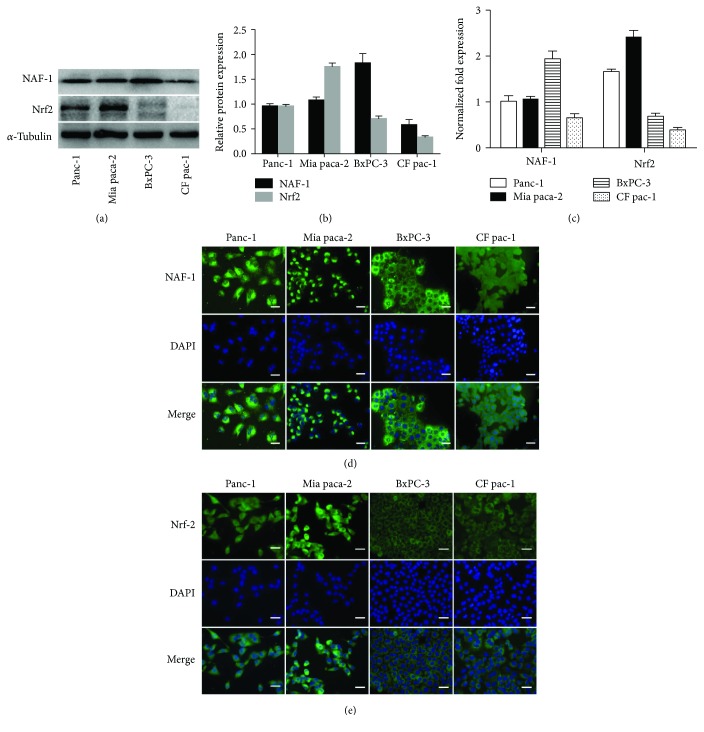
The expression levels of NAF-1 and Nrf2 in four different pancreatic cancer cell lines. (a, b) The expression of NAF-1 and Nrf2 at the protein level in the Panc-1, Mia paca-2, BxPC-3, and CF pac-1 cell lines was detected by Western blotting. The quantified histograms of the Western blots were shown alongside. (c) The normalized fold expression of NAF-1 and Nrf2 at the mRNA level was estimated in four pancreatic cancer cell lines by qRT-PCR. Data are presented as the mean ± SD of three independent experiments. The bar graph shows the relative mRNA expression levels in the cell lines. Column: mean; bar: SD. (d, e) Immunofluorescence staining of NAF-1 and Nrf2 was performed to show the basic expression levels and the locations of the proteins in the cells. NAF-1 and Nrf2 staining is shown in green, and nuclear DNA staining by DAPI is shown in blue. Images are representative of three independent experiments (magnification, 400x; scale bar, 20 *μ*m). Column: mean; bar: SD.

To explore the role of NAF-1 in pancreatic cancer and its relationship with Nrf2, we first examined the expression levels of NAF-1 and Nrf2 in four different pancreatic cancer cell lines (Panc-1, Mia paca-2, BxPC-3, and CF pac-1) by Western blotting, real-time PCR, and immunofluorescence (Figures 2(a)–2(e)). We found marked NAF-1 levels in all four pancreatic cancer cell lines, but weaker in CF pac-1. The expression and transcription of Nrf2 in the Panc-1 and Mia paca-2 lines were strong, but those in the BxPC-3 and CF pac-1 cells were dramatically weaker. Of the four cell lines, Mia paca-2 had the highest Nrf2 expression level. The immunofluorescence showed that NAF-1 is mainly localized in the cytoplasm of pancreatic cancer cells, while Nrf2 is obviously expressed in the cytoplasm and nucleus (Figures 2(d) and 2(e)). Therefore, we chose Panc-1 and Mia paca-2 for the subsequent experiments.

### 3.2. Resveratrol Inhibits Proliferation and Promotes Apoptosis in Pancreatic Cancer Cells

**Figure 3:**
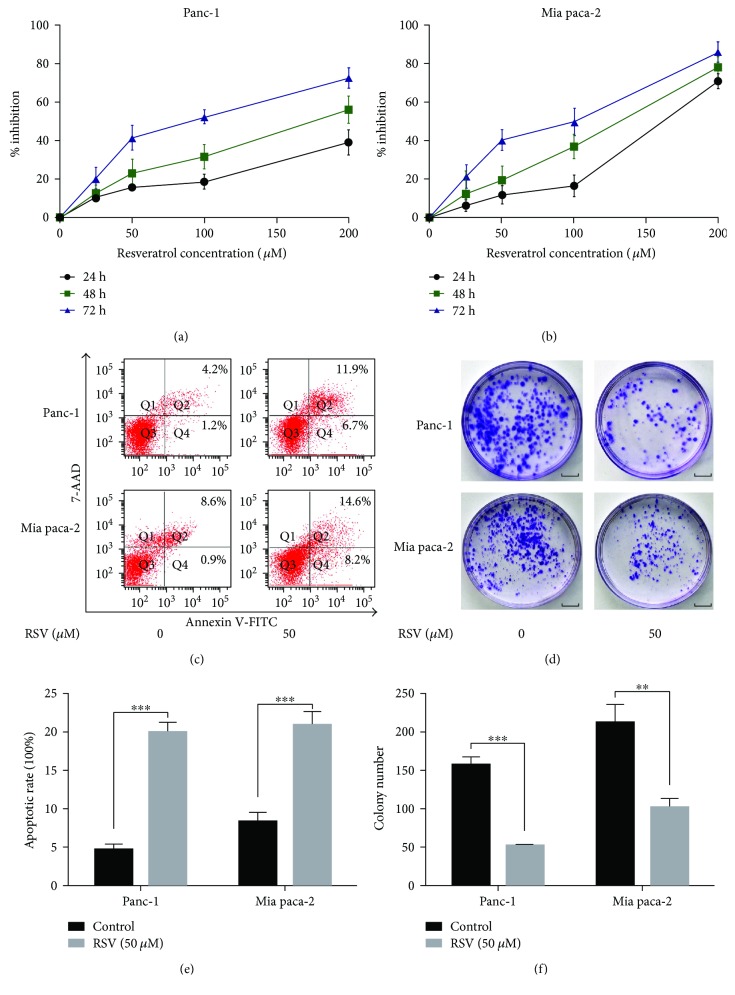
Resveratrol inhibits proliferation and promotes apoptosis in pancreatic cancer cells. (a, b) Two PCa cells, that is, Panc-1 and Mia paca-2, were treated with increasing doses of RSV (0, 25, 50, 100, and 200 *μ*M) for 24 h, 48 h, and 72 h and subjected to an MTT assay for the assessment of the cell viability. (c, e) Flow cytometry was performed to detect the effects of resveratrol on apoptosis in the Panc-1 and Mia paca-2 cells. (d, f) The effects of resveratrol on the colony-forming ability of Panc-1 and Mia paca-2 cells. Images are representative of three independent experiments (scale bar: 1 cm). Column: mean; bar: SD. ^∗∗∗^ *P* < 0.01 and ^∗∗^ *P* < 0.05.

To examine the effects of resveratrol (RSV) on the viability of cancer cells, the pancreatic cancer cells Panc-1 and Mia pac-2 were treated with increasing doses of RSV (0, 25, 50, 100, and 200 *μ*M) for 24 h, 48 h, and 72 h, and the cell viability was assessed using the MTT assay (Figures 3(a) and 3(b)). Resveratrol decreased the growth of the cancer cells in a dose- and time-dependent manner. The low concentrations of RSV (25 and 50 *μ*M) exhibited only slight cytotoxicity, but the high concentrations (100 and 200 *μ*M) impeded the cell viability. These results were consistent with our previous results. Therefore, we chose 50 *μ*M resveratrol for the subsequent experiments. To further explore the inhibitory effect of resveratrol on the proliferation and apoptosis in pancreatic cancer cells, we measured the resveratrol-induced apoptosis in the Panc-1 and Mia paca-2 cells by flow cytometry. The cells were treated with RSV (50 *μ*M) for 48 h, and flow cytometric analyses were conducted in the Panc-1 and Mia paca-2 cells. We found that the 50 *μ*M resveratrol caused an increase in the apoptotic population compared with that in the untreated control cells (Figures 3(c) and 3(e)). Moreover, the treatment with 50 *μ*M resveratrol significantly reduced the colony formation of the Panc-1 and Mia paca-2 cells compared with those of the control groups (Figures 3(d) and 3(f)). These results showed that resveratrol has a potent effect against clone formation and induces apoptosis in cancer cells.

### 3.3. Resveratrol Inhibits the Expression of NAF-1 in Pancreatic Cancer Cells by Activating the Nrf2 Pathways

**Figure 4:**
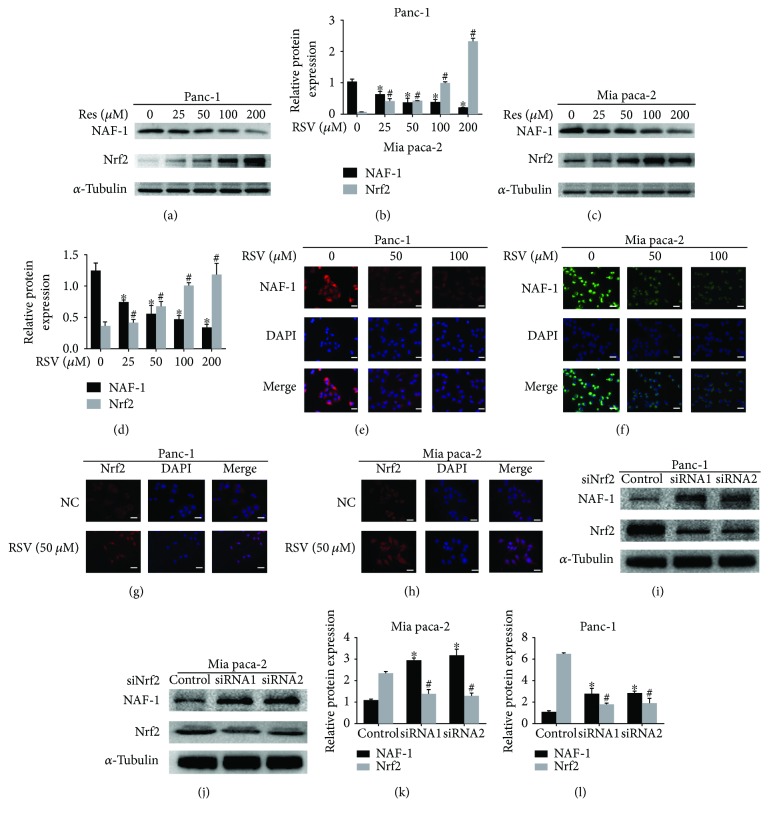
Resveratrol inhibits the expression of NAF-1 in pancreatic cancer cells via activating the Nrf2 pathways. (a–d) The effects of gradient concentrations of resveratrol on the protein expression levels of NAF-1 and Nrf2 were examined by a Western blot analysis using *α*-tubulin as an internal loading control; quantified histograms of the western blots were shown alongside. (e, f) Immunofluorescence staining of NAF-1 in Panc-1 and Mia paca-2 cells was performed to show the effects of resveratrol on the protein expression of NAF-1 after treatment with resveratrol (50 *μ*M and 100 *μ*M) for 24 h. (g, h) Immunofluorescence staining of Nrf2 showed the expression level and translocation of Nrf2 under the resveratrol stimulation in Panc-1 and Mia paca-2 cells (magnification, 400x; scale bar, 20 *μ*m). (i–k) After transfection with siNrf2 or siControl for 48 h, the total proteins in the Panc-1 and Mia paca-2 cells were collected, and the protein expression levels of NAF-1 and Nrf2 were examined by a Western blot analysis using *α*-tubulin as an internal loading control and quantified histograms of the western blots were shown alongside. Column: mean; bar: SD. ^∗^ *P* < 0.05 compared with NAF-1 control group. ^#^ *P* < 0.05 compared with control group.

As reported in the literature, the suppression of NAF-1 results in the activation of apoptosis, prevention of tumors formation, and suppression of tumor growth [4]. In contrast, the overexpression of NAF-1 may promote the proliferation and tumorigenicity of tumors, potentially representing a novel therapeutic target for pancreatic cancer treatments. To determine whether resveratrol affects the expression of NAF-1 in cancer cells, Panc-1 and Mia paca-2 cells were treated with RSV for 48 h and subjected to a Western blot assay to evaluate the effect of RSV on the expression of NAF-1. The images representing the expression of NAF-1 and Nrf2 are derived from the same blot (Figures 4(a) and 4(c)). The results demonstrated that the expression levels of Nrf2 increased, while the level of NAF-1 was significantly inhibited by RSV in a dose-dependent manner. Subsequently, to detect the expression of NAF-1 in response to the treatment with resveratrol, an immunofluorescence assay was performed. The results showed that the NAF-1 levels were downregulated after treatment with different doses of resveratrol (50 *μ*M and 100 *μ*M) and the suppression effect of the higher dose was significantly superior to that of the lower dose (Figures 4(e) and 4(f)). Moreover, immunofluorescence analyses indicated that resveratrol stimulation markedly increased the Nrf2 immunofluorescence signal in both the cytoplasm and the nucleus and induced translocation of Nrf2 to the nucleus (Figures 4(g) and 4(h)). Additionally, to further determine the relationship between NAF-1 and Nrf2, siRNA technology was developed to knock down the expression of Nrf2. The results indicated that downregulation of Nrf2 resulted in the upregulation of the expression level of NAF-1(Figures 4(i) and 4(j)). Quantified analysis of Western blot in Figures 4(a), 4(c), 4(i), and 4(j) was shown alongside the images.

### 3.4. Resveratrol Suppresses the Level of NAF-1 and Enhances the Expression of Nrf2 by Inducing the Accumulation of ROS, Which Contribute to Cell Death in Panc-1 and Mia paca-2 Cells

Resveratrol serves as a protective or therapeutic agent and was shown to exert an antitumor effect in several cancer models. However, in recent years, people pay more attention to the antioxidant activities of resveratrol and ignore the prooxidant activity in cancer cell. Many other in vitro and clinical studies have been conducted involving anticancer activity of resveratrol via increasing intracellular reactive oxygen species (ROS) production. In this study, we aimed to investigate the mechanism of resveratrol in cell death and provide novel evidence to enhance our understanding of the biological property of resveratrol. The cells were treated with increasing doses of RSV (0, 50, 100, 150, and 200 *μ*M) for 24 h, and the intracellular ROS levels were detected using 2′-7′-dichlorodi-hydrofluorescein diacetate (DCFH-DA) probes (Figures 1(a)–1(d)). The results demonstrated that the intracellular ROS levels increased following the treatment with resveratrol. To further explore the downstream mechanism, we treated pancreatic cancer cells with RSV at 50 *μ*M or NAC at 10 mM, respectively, and RSV 50 Μm for 24 h prior to the 24 h incubation of NAC at 10 mM. Protein expression of cancer cells were analyzed using Western blot. The images representing the expression of NAF-1 and Nrf2 are derived from the same blot. The results shown in Figures 1(e) and 1(f)) indicate that H_2_O_2_ (ROS) and RSV treatment significantly improved the expression of Nrf2 and suppressed the level of NAF-1 in the Panc-1 and Mia paca-2 cells. Moreover, NAC, which is a ROS scavenger, significantly reversed the resveratrol-induced suppression of NAF-1 and Nrf2 in the pancreatic cancer cells. Immunofluorescence assay also provided evidence that Nrf2 was activated and transferred into the nucleus increasingly after the resveratrol and H_2_O_2_ treatments (Figures 1(i) and 1(j)). This finding suggests that resveratrol-induced ROS might exert antitumor activity. Therefore, we further examined the effects of ROS production on the expression of apoptosis-related proteins. We found that resveratrol and H_2_O_2_ markedly promoted the expression levels of Bax and suppressed the Bcl-2 level, which are proapoptotic and antiapoptotic molecules in pancreatic cancer cells [37], respectively. NAC, which is a ROS scavenger, could significantly rescue this effect (Figures 1(e) and 1(f)). These results demonstrated that RSV could markedly promote cell death by increasing ROS accumulation and the elevated expression levels of Nrf2 and the downregulation of NAF-1 contributed to the effect of the increased ROS. Then, we aimed to further determine the role of Nrf2 in the inhibition of resveratrol-induced NAF-1. After the transfection with siNrf2 or siControl for 48 h, the Panc-1 and Mia paca-2 cells were treated with resveratrol for 24 h (Figures 1(k) and 1(l)). The protein expression levels of NAF-1, Nrf2, Bax, and Bcl-2 were examined by a Western blot analysis. Interestingly, the results showed that resveratrol could significantly promote the cancer cell death via sequentially inducing Nrf2 upregulation and NAF-1 downregulation, while the siRNA designed for Nrf2 markedly blocked the proapoptosis effect induced by resveratrol. Thus, we demonstrated that resveratrol could promote pancreatic cancer apoptosis through the ROS/Nrf2/NAF-1 pathway. All the proteins detected in Figures 4, 4(f), 4(k), and 4(l) are reprobed from the same blot, respectively.

### 3.5. Knockdown of NAF-1 Increases the Sensitivity of Pancreatic Cancer Cells to Gemcitabine

**Figure 5:**
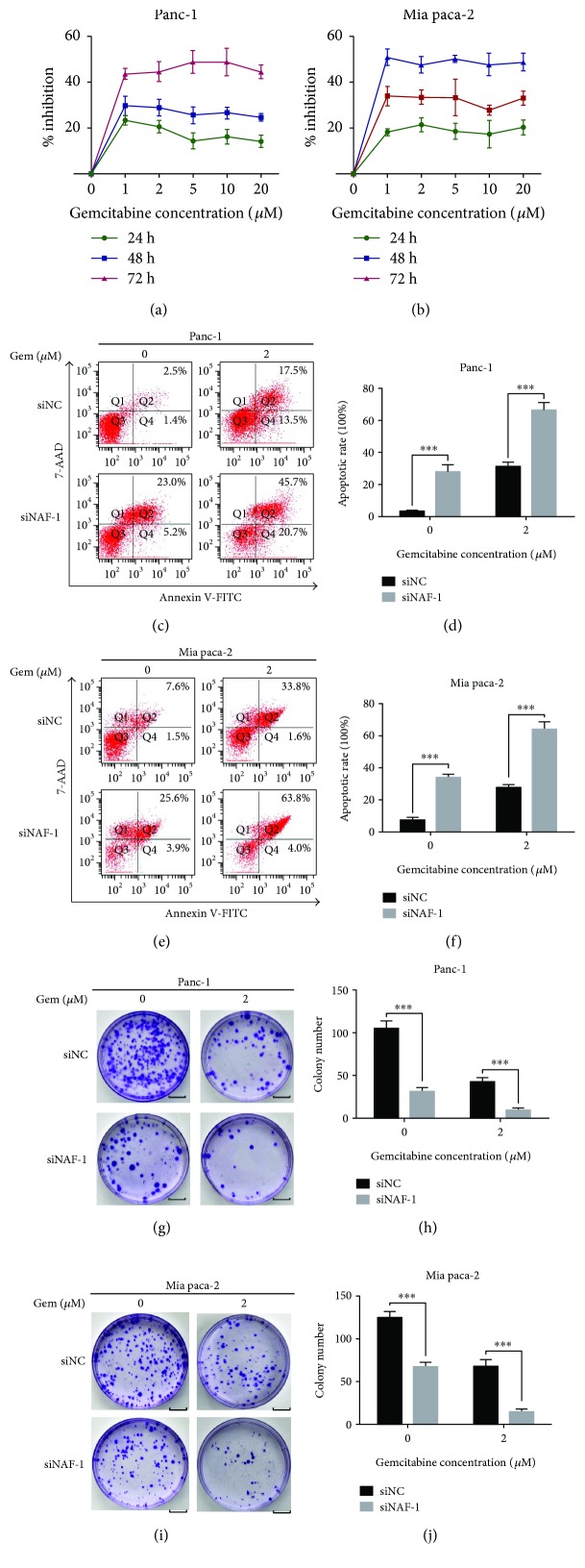
Knockdown of NAF-1 increases the sensitivity of pancreatic cancer cells to gemcitabine. (a, b) After being treated with increasing concentrations of gemcitabine (0, 1, 2, 5, 10, and 20 *μ*M) for 24 h, 48 h, or 72 h, the inhibition ratio of the cancer cell proliferation was detected using an MTT assay. (c–f) The effects of siNAF-1 combined with gemcitabine or siControl on Panc-1 and Mia paca-2 cancer cell apoptosis after a treatment with 2 *μ*M gemcitabine for 48 h; apoptosis in the cancer cells was detected by flow cytometry. (g–j) The effects of siNAF-1 or siControl combined with gemcitabine on the colony-forming ability of the two cell lines were determined using a colony formation assay. Images are representative of three independent experiments (scale bar: 1 cm). Column: mean; bar: SD. ^∗∗∗^ *P* < 0.01.

Here, we aimed to explore the role of NAF-1 in the chemotherapy-increased sensitivity effect in the Panc-1 and Mia paca-2 cell lines. As shown in Figures 5(a) and 5(b), we first detected the effects of gemcitabine on the proliferation of Panc-1 and Mia paca-2 cells via an MTT assay. The apoptotic rate in the two cell lines indicated that the growth of cancer cells was reduced by gemcitabine in a dose- and time-dependent manner. The viability of the pancreatic cancer cells was not obviously impeded with treatment for short period. Both the Mia paca-2 and Panc-1 cells showed an insensitivity to gemcitabine, which was consistent with previous findings [37, 38]. Therefore, we chose 2 *μ*M gemcitabine for the subsequent experiments. To further determine the effects of NAF-1 on cell survival and the response to chemotherapy, we treated the Panc-1 and Mia paca-2 cells, which express high levels of NAF-1 natively and are resistant to gemcitabine, with 2 *μ*M gemcitabine after the transfection with siNAF-1 or siControl for 48 h. We tested the apoptotic rate in the two cell lines using a flow cytometry assay. Our results demonstrated that gemcitabine alone showed minimal effect but gemcitabine along with the siNAF-1 significantly promoted the apoptosis compared with the control group in Panc-1 (Figures 5(c) and 5(d)) and Mia paca-2 cells (Figures 5(e) and 5(f)). The colony formation was significantly decreased after the silencing of NAF-1, and siNAF-1 enhanced the gemcitabine inhibition effect on the cloning ability (Figures 5(g)–5(j)). Altogether, these data suggest that the silencing of NAF-1 enhances the sensitivity of gemcitabine-resistant pancreatic cancer cells to gemcitabine.

### 3.6. Resveratrol-Induced Inhibition of NAF-1 Enhances the Sensitivity of Pancreatic Cancer Cells to Gemcitabine

**Figure 6:**
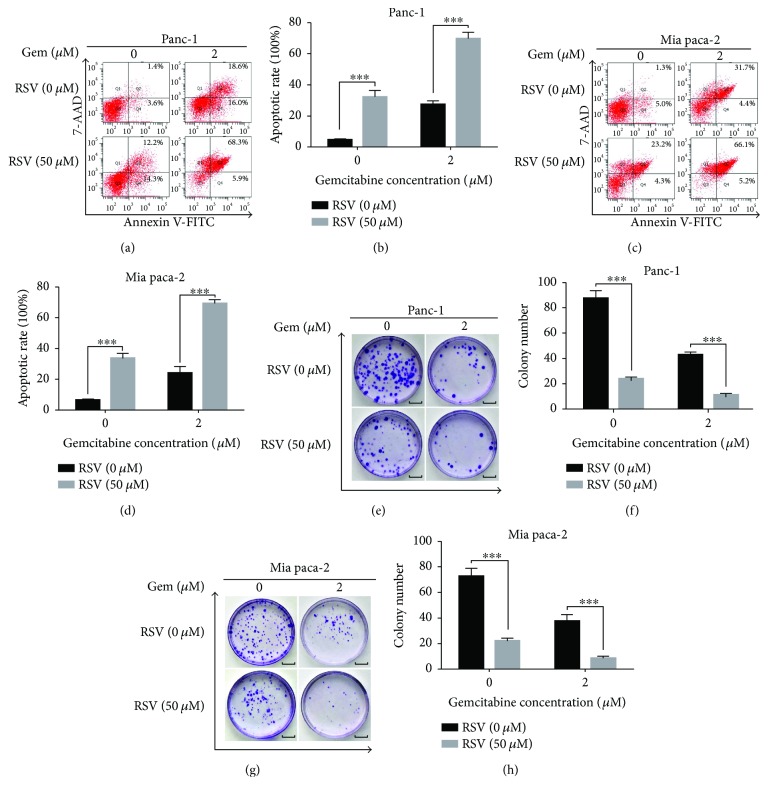
Resveratrol-induced inhibition of NAF-1 enhances the sensitivity of pancreatic cancer cells to gemcitabine. (a–c) Panc-1 and Mia paca-2 cells were treated with gemcitabine (2 *μ*M) and resveratrol (50 *μ*M) for 48 h. Apoptosis was detected by flow cytometry. (e–g) The combined effects of gemcitabine and resveratrol on the colony-forming ability of Panc-1 and Mia paca-2 cells were detected using a colony formation assay. Images are representative of three independent experiments (scale bar: 1 cm). Column: mean; bar: SD. ^∗∗∗^ *P* < 0.01.

To determine whether the resveratrol-induced inhibition of NAF-1 increases the susceptibility of pancreatic cancer cells to gemcitabine, we treated the Panc-1 and Mia paca-2 cells with a combination of 50 *μ*M resveratrol and 2 *μ*M gemcitabine, and the MTT assay was performed. The results indicated that the combination of gemcitabine and resveratrol significantly promoted the rate of apoptosis (Figures 6(a)–6(d)). This finding suggests that resveratrol, accompanied with gemcitabine, showed obvious inhibitory effects on apoptosis and growth. To further confirm this result, we sequentially tested the proliferation of pancreatic cells using a colony formation assay. Similarly, the combination of gemcitabine and resveratrol was significantly more effective than gemcitabine or resveratrol treatment alone (Figures 6(e)–6(g)). Altogether, these results demonstrated that resveratrol has a potent effect in enhancing the sensitivity of pancreatic cancer cells to gemcitabine by inhibiting NAF-1 expression.

## 4. Discussion

In our present study, we investigated whether resveratrol has a potentially protective effect on pancreatic cancer. To clarify the mechanism of resveratrol in pancreatic cancer, we performed a series of experiments and detected the impact induced by resveratrol on downstream signaling molecules. Our results showed that resveratrol could activate Nrf2 and suppress the expression NAF-1 in pancreatic cancer cells by inducing the accumulation of ROS. More importantly, decreasing the level of NAF-1 impeded the proliferation of pancreatic cancer cells and activated apoptosis. Targeting NAF-1 via resveratrol can enhance the sensitivity of pancreatic cancer cells to gemcitabine. Optimistically, we further explored the role of NAF-1 as a novel molecular target for improving the efficacy of the current chemotherapeutic regimens used in patients with pancreatic cancer and improving their clinical prognosis.

Although many studies have been performed and the mechanisms underlying chemotherapy resistance in pancreatic cancer have been explored, a low response rate to gemcitabine is common in the clinic, gemcitabine exhibits restricted effects [39], and less than 20% of patients experience the ideal effects of gemcitabine [40]. Recently, a combination of oxaliplatin, leucovorin, fluorouracil, and irinotecan, which is called FOLFIRINOX, has been widely used as a clinical therapy for the treatment of metastatic pancreatic cancer patients [41–43]. However, the FOLFIRINOX treatment program is accompanied by seriously adverse reactions and drug resistance, which limits its cytotoxic efficacy. Therefore, finding a novel target for enhancing the efficiency of chemotherapy is clearly needed to improve the outcomes of patients with pancreatic cancer.

Due to its known antioxidant activity, resveratrol exerts beneficial effects through reducing apoptosis, inflammation, and other oxidative stress-related processes [44]. Recently, the extensive antitumor effects of resveratrol have been revealed, and its functions include inhibiting proliferation, inducing apoptosis, repressing invasion and migration, and impairing the tumor-initiating stem-like properties via several signaling pathways, such as the sonic hedgehog pathway and the PI-3K/Akt/NF-kappaB pathway [45, 46]. Of course, resveratrol also causes apoptosis, autophagy, and ultimately cell death mediated by ROS [16]. It has been demonstrated that many malignant tumor cells are accompanied by elevated levels of cellular ROS, which plays a crucial role in the initiation and progression of cancer by promoting cell proliferation, invasion, and metastasis [13, 20, 36]. In most cases, resveratrol demonstrated antioxidant properties by modulating the activity of antioxidant enzymes, which is thought to contribute to its anticancer effects [47, 48]. For example, some polyphenol materials function as prooxidants and induce the expression of oxidative stress-related genes [49]. Interestingly, in line with the findings that resveratrol is able to promote ROS, our results demonstrated that the treatment with resveratrol increased the intracellular ROS levels in the pancreatic cancer cells. The resveratrol-induced ROS influence the level of NAF-1 by upregulating Nrf2, which protects the pancreatic cancer cells from the gemcitabine therapy. We demonstrate that resveratrol contributes to pancreatic cancer cell death by inducing the accumulation of ROS and suppressing the expression of NAF-1. The discrepancy may be explained by the different dose or duration of resveratrol supplementation and disparates cell condition or environment.

More interestingly, this study is the first to discover the relationship between the NAF-1 and Nrf2 proteins. Nrf2 signaling pathway is usually considered as the most important cellular molecular pathway involving oxidative stress and apoptosis acting as cellular sensors of chemical- and radiation-induced oxidative and electrophilic stress [50–52]. In this study, the specific role of Nrf2 as a tumor suppressor in pancreatic cancer cells is explored. As a tumor promoter, Nrf2 is usually upregulated, thus helping malignant cells to withstand high levels of ROS and avoid apoptosis through activation of metabolic and cytoprotective genes that contribute to enhanced cell proliferation [53, 54]. When the cancer cells are exposed to oxidative stress, Nrf2 pathway is switched on, which is closely related to cell proliferation, apoptosis, and drug resistance [55, 56], and thus helping malignant cells to withstand high levels of ROS and avoid apoptosis through activation of metabolic and cytoprotective genes that contribute to enhanced cell proliferation [53, 54]. However, more recent data focus on its role also as a tumor suppressor [32, 33]. RTA 405, an antioxidant inflammation modulator, as well as resveratrol, mediates activation of Nrf2 and does not promote growth or survival of cancer cells. Probst et al. have also found that RTA 405 suppresses cancer cell survival and promotes apoptosis via downregulating the NF-*κ*B activity. Resveratrol may have the same pharmacology action and mechanism to promote the apoptosis of pancreatic cancer cells via activation of Nrf2 pathway.

In this study, we found that the Nrf2 pathway and NAF-1 negatively interact with one another upon ROS stimulation, which has a crucial importance in promoting pancreatic cancer cell death. As we know, the relationship between Nrf2 pathway and autophagy has been explored in many years. Many studies have revealed a previously unappreciated role of Nrf2 pathway in the regulation of autophagy and make a consistent relation [31, 57, 58]. Nrf2 pathway and autophagy have a parallel interaction with each other in certain conditions. In this study, we put forward ideas that autophagy probability serves as a key role in putting them into context of their relationship. Moreover, NAF-1 functions as an important regulator of intracellular autophagy and serves as an important regulator by antagonizing the BECN1-mediated cellular autophagy at the endoplasmic reticulum [59]. Therefore, we believe that the activation of Nrf2 pathway induced by resveratrol results in the activation of autophagy and then the downregulation of NAF-1. But it still needs more work to explore the exact mechanisms between NAF-1 and Nrf2 pathway.

Because NAF-1 is important for lifespan control and autophagy, NAF-1 is associated with proliferation and apoptosis in breast cancer [4, 11]. Bai et al. have reported that NEET proteins are a novel class of drug targets in the chemotherapeutic treatment of breast cancer and that MAD-28 can be used as a template for rational drug design for NEET Fe-S cluster-destabilizing anticancer drugs [60]. However, its role in pancreatic cancer, particularly in drug resistance in cancer, has not been well studied. Our study adds to the accumulating evidence suggesting that NAF-1 can improve the chemotherapeutic sensitivity of pancreatic cancer cells and improve the clinical effectiveness of drugs. Resveratrol, which served as a nonspecific inhibitor of NAF-1, may exhibit greater efficacy and lower toxicity in the prevention and treatment of pancreatic cancer. Next, we aim to explore the downstream molecules in drug resistance in pancreatic cancer and hope to discover more specific mechanisms.

## 5. Conclusions

In conclusion, the present study demonstrated that resveratrol suppressed the proliferation and cloning ability and induced the apoptosis of pancreatic cancer cells. These multiple biological effects might result from the negative interaction between Nrf2 and NAF-1 upon ROS accumulation. And ROS production induced by resveratrol not only leads to activation of Nrf2 but also inhibits NAF-1 transcriptional activity. The demonstrated relationship between Nrf2 and NAF-1 will further advance our understanding of the progression of pancreatic cancer induced by ROS. NAF-1 silenced by siRNA or resveratrol could enhance the sensitivity of gemcitabine in pancreatic cancer cells. Thus, novel drug targets for inhibition of NAF-1 may be a potential therapy for preventing the progression of pancreatic cancer.

## Supplementary Materials

Supplementary Materials Table S1: a list of the utilized primary antibodies. Table S2: the siRNA sequences. Table S3: primer sequences for real-time PCR analysis.
