# Supplementary material for: Resveratrol-Induced Downregulation of NAF-1 Enhances the Sensitivity of Pancreatic Cancer Cells to Gemcitabine via the ROS/Nrf2 Signaling Pathways
Source: Oxid Med Cell Longev. 2018 Mar 22;2018:9482018. doi: 10.1155/2018/9482018 (PMC5885341; doi:10.1155/2018/9482018)
Supplement: Supplementary Materials — Table S1: a list of the utilized primary antibodies. Table S2: the siRNA sequences. Table S3: primer sequences for real-time PCR analysis. [file 9482018.f1.pdf]

Resveratrol-Induced Down-Regulation of NAF-1 Enhances the Sensitivity of Pancreatic Cancer Cells to Gemcitabine via the ROS/Nrf2 Signaling Pathways

Liang Cheng, Bin Yan, Zhengdong Jiang, Ke Chen, Cancan Zhou, Junyu Cao, Weikun Qian, Jie Li, Liankang Sun, Qingyong Ma and Huanchen Sha

Table 1. A list of the utilized primary antibodies.

Antibody	Dilution & Use	Company
Rabbit anti-NAF-1	1:1000 (WB)<br>1:200 (IF)	Abcam
Rabbit anti-Nrf2	1:1000 (WB)	Abcam
Rabbit anti-Bax	1:2000 (WB)	Abcam
Rabbit anti-Bcl-2	1:2000 (WB)	Abcam
Mouse anti-α-tubulin	1:10,000 (WB)	Proteintech
Goat anti-rabbit IgG-HRP	1:10,000 (WB)	Abbkine. Inc
Goat anti-rabbit dylight 594 (red) IgG antibody	1:200 (IF)	Abbkine. Inc

Table 2. The siRNA sequences.

Genes	Primer Sequences
siControl	sense: 5′-UUCUCCGAACGUGUCACGUTT-3′<br>antisense: 5′-ACGUGACACGUUCGGAGAATT-3′
siNAF-1	sense: 5′- CCUGAAAGCAUUACCGGGUUGGCGCUA-3′<br>antisense: 5′-UAGCGAACCCGGUAAUGCUUUCAGC-3′
siNrf2	sense: 5′-UUCUCCGAACGUGUCACGUTT-3′<br>antisense: 5′-ACGUGACACGUUCGGAGAATT-3′

Table 3. Primers sequences for real-time PCR analysis.

Genes	Primer Sequences
NAF-1	Forward: 5′-GCAAGGTAGCCAAGAAGTGC-3′<br>Reverse: 5′CCCAGTCCCTGAAAGCATTA-3′
Nrf2	Forward: 5′-GGAGGCAAGAUAUAGAUCUTT-3′<br>Reverse: 5′-AGAUCUAUAUCUUGCCUCCTT-3′
β-actin	Forward: 5′-AGCGAGTATCCCCCAAAGTT-3′<br>Reverse: 5′-GGGCACGAAGGCTCATCATT-3′
